# Prevalence of Sexually Transmitted Infections and Predictors for Loss to Follow Up among Marginalized Homeless and Migrant Communities: a Cross-Sectional Study

**DOI:** 10.5334/aogh.4388

**Published:** 2024-04-01

**Authors:** Francesco Vladimiro Segala, Roberta Novara, Gianfranco Panico, Renato Laforgia, Lucia Raho, Marcella Schiavone, Giovanni Civile, Nicole Laforgia, Stefano Di Gregorio, Giacomo Guido, Mariangela Cormio, Angelo Dargenio, Roberta Papagni, Angelo L’Erario, Luca L’Erario, Valentina Totaro, Vito Spada, Lauriana Valentini, Luisa Frallonardo, Rossana Lattanzio, Carmine Falanga, Giovanni Putoto, Annalisa Saracino, Francesco Di Gennaro

**Affiliations:** 1 Clinic of Infectious Diseases, Department of Precision and Regenerative Medicine and Ionian Area (DiMePRe-J), University of Bari Aldo Moro, 70124 Bari, Italy; 2 Doctors with Africa CUAMM, 70123 Bari, Italy; 3 Diagnostic Laboratory: Laboratorio analisi Dott.ssa Dell’Olio Nunzia, Trani, Italy; 4 Department of Drug Chemistry and Technologies, Sapienza University of Rome, P.le A. Moro 5, 00185, Rome, Italy; 5 ANLAIDS Sezione Lombarda, 20124 Milano, Italy

**Keywords:** STI, homeless, migrants, screening, return rate, healthcare

## Abstract

**Background:**

In Europe and Italy, marginalized communities have a higher risk for both contracting sexually transmitted infections (STI) and progressing towards adverse outcomes.

**Objectives:**

This study focuses on the screening of HIV, HBV, HCV, and syphilis among homeless individuals and agricultural migrant workers living in Apulia, Italy. It aims to assess STI prevalence and investigate factors that might hinder return to collect test results. In addition, it explores STI knowledge, attitudes, and practices among these vulnerable populations.

**Methods:**

A cross-sectional study was conducted from September 1, 2022, to September 30, 2023. Participants were recruited from community health centers and migrant camps. Blood tests for HBV, HCV, HIV, and syphilis were performed, and Knowledge, Attitude, and Practices (KAP) survey were conducted via face-to-face interviews. Descriptive and logistic regression analyses were used to assess factors influencing the return for test results.

**Results:**

A total of 149 persons were recruited, including 64 agricultural migrant workers and 85 homeless people. Overall, 24.8% (n = 37) tested positive for at least one infection, and only 50.3% (n = 75) of the screened participants returned to collect their test results. Significant disparities in STI knowledge and healthcare access were observed between the two populations, with only 14.1% (n = 9) of migrants having access to primary healthcare. At multivariable analysis, the strongest predictor for not returning for test results was being positive for HCV.

**Conclusions:**

Among homeless people and agricultural migrant workers, STI prevalence was high, and only half of the population returned to collect test results. The study underscores the urgent need for targeted interventions and policy reevaluation to address healthcare disparities in marginalized communities.

## Background

Despite the concerted efforts in Europe and Italy to eradicate discrepancies in healthcare, substantial gaps in disease burden persist in disadvantaged populations, disparities that worsened during the COVID-19 pandemic [1]. Individuals living in poverty are significantly more prone to experiencing healthcare inequities due to a limited access to public health system and longer diagnostic delays, resulting in more severe clinical outcomes and overall worse prognosis [2,3]. In this scenario, both undocumented migrants and homeless people constitute a highly vulnerable population, with their health being significantly influenced by social determinants such as housing, poverty, and education [4]. Specifically, these populations are deemed to be particularly at high risk for sexually transmitted infections (STI), including human immunodeficiency virus (HIV), hepatitis B (HBV), and hepatitis C (HCV) [3].

Given that these infections are characterized by a prolonged asymptomatic phase, many individuals with HIV, HBV, and HCV are unaware of their condition. In the year 2020, within the WHO European Region, a total of 2.6 million people were living with HIV. Among them, 77% were aware of their HIV status, 83% of those diagnosed were on antiretroviral therapy (ART), and 95% of individuals receiving ART achieved viral suppression [5]. Also, it is estimated that only one fifth of people currently living with HBV and a quarter of people living with HCV are diagnosed, with higher rates of undiagnosed infections among vulnerable populations [6,7].

As a consequence, continuous STI surveillance in marginalized communities is a key step in the pursuit of United Nations Sustainable Development Goals [8] and in the optimization community-based preventive and therapeutic strategies. Specifically, even within the context of a well-designed screening program, it is well described that, in under-resourced communities, a significant challenge is represented by linkage and retention in care [9].

In line with these objectives, we aimed to assess the rate of return for test results (RTR) after screening for HIV, HBV, HCV, and syphilis in homeless people and agricultural migrant workers living in the Apulia region of Italy. Furthermore, we aimed to assess the prevalence of these infections and to explore STI knowledge, attitude, and practices (KAPs) about STIs among these two vulnerable populations.

## Methods

### Study design, population and setting

We performed a cross-sectional study from September 1, 2022, to September 30, 2023. Recruitment and data collections were performed by a multidisciplinary group comprising an infectious diseases specialist, an infectious diseases medical resident, two nurses, one cultural mediator, and one volunteer healthcare worker affiliated with the Italian non-governmental organization Doctors with Africa CUAMM (University College for Aspiring Medical Missionaries). This organization has been actively engaged in assisting the agricultural migrant worker communities since 2015. STI and KAP screening surveys were delivered concurrently by face-to-face interviews.

Study sites included four community health centers for homeless people situated in the City of Bari and five resettlement camps located in the municipality of Foggia, so-called ‘ghettos,’ where agricultural migrant workers (AMW) reside. A more detailed description of sites included in the study is provided in the supplementary file.

The eligible population included all adult individuals who were beneficiaries or residents in the mentioned facilities throughout the study period. No exclusion criteria were applied for this study. Participants had the option to withdraw from the study at any time, and their decision did not impact their medical care.

Blood test result delivery was scheduled approximately 10 days after screening, and the failure to return for results rate (FTR) was assessed. Participants who tested positive for any of the infections included in the study were referred for further diagnostic evaluation and treatment to the Clinic of Infectious Diseases of the Polyclinic of Bari. Positive-screened participants received the same standard of care, in accordance with national and international guidelines [10,11,12,13].

### Questionnaire development and data collection

Questionnaire development was informed by literature review and administered through face-to-face interviews conducted by nurses and trained doctors. Every interaction with the subjects involved in the study was facilitated by linguistic and cultural mediators, when needed. To minimize the social desirability bias, interviewers provided clear instructions at the beginning of the questionnaire, explaining the purpose of the survey, the importance of honest responses, and reassuring participants about data confidentiality.

The questionnaire was organized into the following sub-sections:

– Socio-demographic information (age, education, occupation, marital status, how long they were homeless, possible employment contract, residence document in Italy): 6 questions.
– Risk factors for STI: multiple sexual partners, use of condoms, smoking and alcohol habits, chem-sex (defined as “voluntary intake of psychoactive drugs with the intention of facilitating and/or enhancing sexual activity [14]”) and medical history (including symptoms of TB and other communicable-non-communicable diseases): 6 questions.
– Knowledge, attitude, and practices on HIV, HBV, HCV, and Syphilis: 25 questions.

All study participants underwent the following blood tests:

– Serologies for HBV (HBsAg, anti-HBs, and anti-HBc), HCV (anti-HCV), human immunodeficiency virus (HIV), and syphilis (VDRL and TPHA).
– The study participants, resulting positive for the screening, performed confirmation tests according to the standard of care: HBV-DNA for HBV, HCV-RNA and genotype for HCV, HIV-RNA, immune cell count, and resistance profile for HIV.

All data were entered into a secured online platform (Kobo Ttoolbox [15]), and a quality control check of the data entry was performed before data analysis.

### Statistical analysis

A descriptive analysis was performed to define the distribution of the characteristics of the sample. Continuous data were summarized as median and interquartile range (IQR). Return For Test Results (RTR) was identified as the dependent variable. World regions were classified according to the United Nation Geoscheme [16]. The distribution of continuous variables was assessed with the Shapiro-Wilk normality test, and the Mann-Whitney U test was used to compare groups for continuous variables. A chi-squared test (with the Fisher’s correction if less than five cases were present in a cell) was applied for categorical variables. A logistic regression model was implemented as follows. RTR was considered as a dependent variable and each of the available factors at the baseline evaluation as independent variables (univariate analysis). The effect sizes were reported as odds ratio (OR) with 95% confidence interval (CI). All tests were 2-sided, and a p-value <0.05 was considered statistically significant. The model included a set of clinically relevant candidate predictors and variables resulted to be significant at the bivariate analysis. Statistical analyses were performed using R Statistical Software (v4.1.3; R Core Team 2021) in R Studio Version [17].

### Ethical considerations

Prior to recruitment, written informed consent (prepared in 4 languages: Italian, French, English, and Arabic) was obtained from all participants. The objectives of the study, along with methods used and data confidentiality, were explained with the aid of cultural mediators, when needed. The study has been approved by Ethics Committee ‘Azienda Ospedaliero-Universitaria Consorziale Policlinico,’ protocol number DG1563. During the screening process, we actively worked to establish and enhance personalized pathways for connecting individuals to healthcare through various social and healthcare services. We raised awareness about the potential progression of HIV and liver diseases, as well as the risk of transmission to others, by directing these individuals to the Infectious Disease Clinic at the Policlinic of Bari for counseling, diagnosis, and appropriate treatment.

## Results

### Characteristics of the population

From September 1, 2022, to September 30, 2023, a total of 149 participants were recruited in the study, of which 64 (42.9%) were agricultural migrant workers and 85 (57.1%) were homeless, with a median age of 35 (IQR: 27–45) years. Characteristics of the sample stratified for study subpopulation and RTR are displayed in Table 1. Males accounted for 87.5% (n = 56) of agricultural migrant workers and 75.3% (n = 64) of homeless people. The most frequently reported comorbidities were diabetes (8.1%, n = 12), hypertension (6%, n = 9), and respiratory pathologies (4%, n = 6). Among agricultural migrant workers, countries of origin were located mainly in sub-Saharan Africa (64.1%, n = 41) and North Africa (20.3%, n = 13), while 32.9% (n = 28) and 31.8% (n = 27) of homeless people were originally from Sub-Saharan Africa and Italy. Educational level was low, with 64.4% (n = 96) of the included subjects reporting only primary school or no formal education. Overall, 69.1% (n = 103) of the participants did not have access to primary healthcare, and this rate was alarmingly high among people residing in the Apulian ghettos (85.9% n = 55 vs 56.5% n = 48, p < 0.001). Apart from country of origin (p < 0.001) and access to care, the migrant and homeless subpopulation differed significantly on the following variables: reporting chem-sex (p = 0.049) and HCV and HIV knowledge (p < 0.001 and p = 0.02)—with homeless people scoring better.

**Table 1:** Characteristics of the population stratified for subpopulation and return rate.


	OVERALL (N = 149)	SUB-POPULATION			RETURN FOR TEST RESULTS		
	
		AGRICULTURAL MIGRANT WORKERS (N = 64)	HOMELESS PEOPLE (N = 85)	P-VALUE FOR SETTING	NOT RETURNED (N = 74)	RETURNED (N = 75)	P-VALUE FOR RETURNING

**Age, median [Q1, Q3]**	35 [27, 45]	34 [26, 42]	35 [29, 48]	0.328	33.0 [26.3, 41.8]	36.0 [28.5, 50.0]	0.171

**Gender**							

F	29 (19.5%)	8 (12.5%)	21 (24.7%)	0.0982	11 (14.9%)	18 (24.0%)	0.23
	
M	120 (80.5%)	56 (87.5%)	64 (75.3%)		63 (85.1%)	57 (76.0%)	

**Sub-populations**							

Agricultural migrant workers	64 (43.0%)	–	–	–	38 (51.4%)	26 (34.7%)	0.0585
	
Homeless people	85 (57.0%)	–	–		36 (48.6%)	49 (65.3%)	

**Marital Status**							

Divorced	11 (7.4%)	3 (4.7%)	8 (9.4%)	0.442	3 (4.1%)	8 (10.7%)	0.478
	
In an open relationship	4 (2.7%)	2 (3.1%)	2 (2.4%)		2 (2.7%)	2 (2.7%)	
	
Single	84 (56.4%)	34 (53.1%)	50 (58.8%)		44 (59.5%)	40 (53.3%)	
	
Married	49 (32.9%)	25 (39.1%)	24 (28.2%)		25 (33.8%)	24 (32.0%)	
	
Missing	1 (0.7%)	0 (0%)	1 (1.2%)		0 (0%)	1 (1.3%)	

**Region of origin**							

Eastern Europe	10 (6.7%)	6 (9.4%)	4 (4.7%)	**<0.001**	4 (5.4%)	6 (8.0%)	0.25
	
Middle East	10 (6.7%)	1 (1.6%)	9 (10.6%)		8 (10.8%)	19 (25.3%)	
	
North Africa	24 (16.1%)	13 (20.3%)	11 (12.9%)		4 (5.4%)	6 (8.0%)	
	
Other	2 (1.3%)	1 (1.6%)	1 (1.2%)		14 (18.9%)	10 (13.3%)	
	
Southeast Asia	7 (4.7%)	2 (3.1%)	5 (5.9%)		1 (1.4%)	1 (1.3%)	
	
Sub-Saharan Africa	69 (46.3%)	41 (64.1%)	28 (32.9%)		3 (4.1%)	4 (5.3%)	
	
Italy	27 (18.1%)	0 (0%)	27 (31.8%)		40 (54.1%)	29 (38.7%)	

**Education**							

Primary school	56 (37.6%)	29 (45.3%)	27 (31.8%)	0.0872	33 (44.6%)	23 (30.7%)	0.373
	
Lower secondary school	36 (24.2%)	13 (20.3%)	23 (27.1%)		15 (20.3%)	21 (28.0%)	
	
Higher secondary school	14 (9.4%)	4 (6.3%)	10 (11.8%)		5 (6.8%)	9 (12.0%)	
	
University	3 (2.0%)	3 (4.7%)	0 (0%)		1 (1.4%)	2 (2.7%)	
	
No formal education	40 (26.8%)	15 (23.4%)	25 (29.4%)		20 (27.0%)	20 (26.7%)	

**Comorbidities**							

Diabetes	12 (8.1%)	2 (3.1%)	10 (11.8%)	0.106	4 (5.4%)	8 (10.7%)	0.379

Hypertension	9 (6.0%)	2 (3.1%)	7 (8.2%)	0.343	4 (5.4%)	5 (6.7%)	1

Cardiopathy	4 (2.7%)	0 (0%)	4 (4.7%)	0.212	1 (1.4%)	3 (4.0%)	0.622

Pneumopathy	6 (4.0%)	0 (0%)	6 (7.1%)	0.0803	3 (4.1%)	3 (4.0%)	1

Neuropsychiatric disorder	4 (2.7%)	0 (0%)	4 (4.7%)	0.212	2 (2.7%)	2 (2.7%)	1

Cancer	2 (1.3%)	1 (1.6%)	1 (1.2%)	1	0 (0%)	2 (2.7%)	0.482

TB History	3 (2.0%)	1 (1.6%)	2 (2.4%)	1	1 (1.4%)	2 (2.7%)	1

**Chem sex**	14 (9.4%)	2 (3.1%)	12 (14.1%)	**0.0494**	4 (5.4%)	10 (13.3%)	0.177
	
Missing	1 (0.7%)	1 (1.6%)	0 (0%)		1 (1.4%)	0 (0%)	

**Consistent condom use**	21 (14.1%)	10 (15.6%)	11 (12.9%)	0.819	9 (12.2%)	12 (16.0%)	0.662

**Access to primary healthcare**	46 (30.9%)	9 (14.1%)	37 (43.5%)	**<0.001**	15 (20.3%)	31 (41.3%)	**0.009**

**Fear of community exclusion in case of HIV positivity**	88 (59.1%)	37 (57.8%)	51 (60.0%)	0.92	43 (58.1%)	45 (60.0%)	0.946

**Fear of community exclusion in case of HCV positivity**	38 (25.5%)	16 (25.0%)	22 (25.9%)	1	16 (21.6%)	22 (29.3%)	0.372

**Being tested for HIV or HCV in the past**	38 (25.5%)	14 (21.9%)	24 (28.2%)	0.489	18 (24.3%)	20 (26.7%)	0.889

**HCV Knowledge Score, median [Q1, Q3]**	0 [0, 2]	0 [0, 1]	1 [0, 3]	**0.001**	0 [0, 1]	0 [0, 3]	**0.04**

**HIV Knowledge Score, median [Q1, Q3]**	2 [0, 4]	2 [0, 3]	3 [1, 4]	**0.019**	2 [0, 3]	2 [0, 4]	0.512

**Infections**							

HIV	3 (2.0%)	2 (3.1%)	1 (1.2%)	0.803	1 (1.4%)	2 (2.7%)	1

Syphilis	5 (3.4%)	2 (3.1%)	3 (3.5%)	1	5 (6.8%)	0 (0%)	0.0665

HCV	14 (9.4%)	6 (9.4%)	8 (9.4%)	1	12 (16.2%)	2 (2.7%)	**0.01**

HBV Infection	21 (14.1%)	10 (15.6%)	11 (12.9%)	0.819	11 (14.9%)	10 (13.3%)	0.974

Any infection	37 (24.8%)	17 (26.6%)	20 (23.5%)	0.816	24 (32.4%)	13 (17.3%)	**0.052**

More than one infection	6 (4.0%)	3 (4.7%)	3 (3.5%)	1	5 (6.8%)	1 (1.3%)	0.205

**Vaccinated against HBV**	21 (14.1%)	5 (7.8%)	16 (18.8%)	0.078	7 (9.5%)	58 (77.3%)	0.138
	
Missing	3 (2.0%)	0 (0%)	3 (3.5%)		0 (0%)	14 (18.7%)	

**Returned for test results**	75 (50.3%)	26 (40.6%)	49 (57.6%)	0.058		–	–


### Knowledge, attitudes, and practices towards sexually transmitted infections

Concerning HIV/AIDS, only 60% (n = 90) of the recruited individuals were aware that the disease can be transmitted through sexual contact. Additionally, 45.7% (n = 65) were not aware of the protection offered by condoms. A notable 34.9% (n = 52) agreed or strongly agreed that HIV/AIDS might be transmitted by sharing utensils or having a meal together. Moreover, over 75% (n = 111) of the sample did not agree with the statement ‘thanks to HIV treatment, people can lead a high-quality life and not be contagious anymore.’ Awareness of Hepatitis C was notably low, with limited knowledge about its modes of transmission, potential complications, and available treatment options in both subpopulations. Details about the distribution of responses to the KAP questionnaire are provided in **Supplementary Table 1**. Both HIV (p < 0.001) and HCV (p = 0.02) knowledge were significantly lower among agricultural migrant workers (Table 1).

Regarding the perception of stigma, the majority (59.1%, n = 88) of participants expressed fear that a positive HIV test would lead to social exclusion, while only 25.5% (n = 38) held similar concerns about a positive HCV test. In terms of practices, the majority of participants (85.9%, n = 128) reported inconsistent condom use, while 14.1% (n = 12) of homeless individuals stated that they had engaged in chem-sex.

### Sexually transmitted infections screening

**Figure 1:**
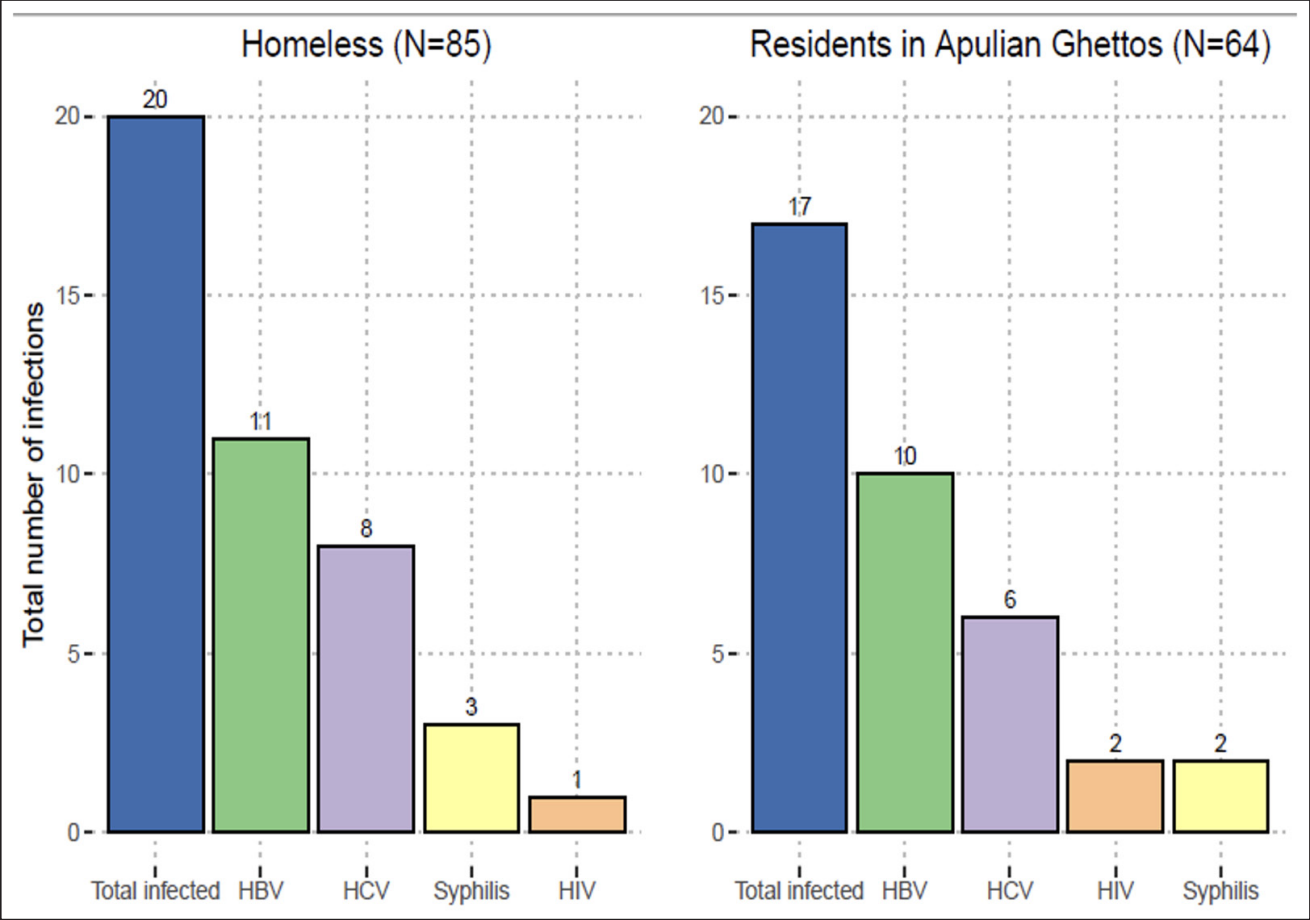
Distribution of infections among migrants and homeless people.

**Figure 2:**
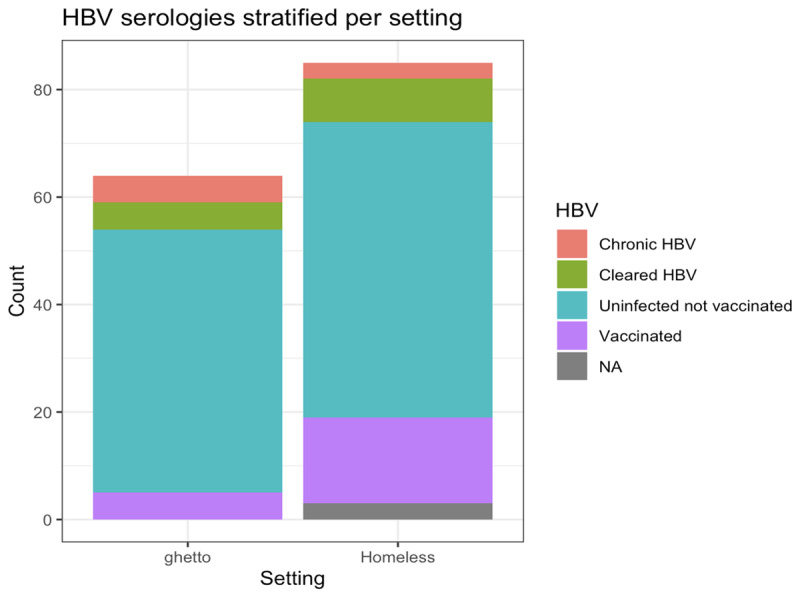
HBV serologies stratified per subpopulation.

The total number of infections detected is illustrated in Figure 1. Overall, out of 149 participants screened, 3 tested positive for HIV (2%), 14 for HCV (9.4%), 21 (14.1%) for HBV, and 5 (3.4%) for syphilis. Only 14% were vaccinated for HBV. HBV serology results (Figure 2) indicate that most of the study participants have neither been vaccinated against HBV nor have they ever been exposed to the virus. Approximately one out of four people (24.8%, n = 37) were positive for at least one infection, while three people out of four (74.5%, n = 111) reported that they had never undergone prior testing for these infections.

### Mono and multivariable analysis for return rate for test results

Overall, only half of the screened participants (50.3%, n = 75) returned to collect test results. As shown in Table 1, at univariate analysis, people who had access to primary healthcare were significantly associated with returning (41.3% n = 31 vs 20.3% n = 15, p < 0.009), as well as people who performed better at HCV knowledge questionnaire (p = 0.04). Alarmingly, people whose results indicated infection with HCV or with at least one STI were more likely to be lost to follow-up.

At multivariable analysis, the only factor independently associated with reduced odds of returning to collect test results was being positive to HCV (Table 2).

**Table 2:** Multivariable logistic regression model for return for test results.


FACTOR	aOR	lowCI	highCI	pVALUE

(Intercept)	0.845	0.398	1.775	0.657

**Living in Apulian Ghettos**	0.697	0.332	1.462	0.337

**Having access to primary healthcare**	2.101	0.928	4.881	0.077

**Good HCV knowledge**	2.208	0.911	5.583	0.083

**Having contracted HCV**	0.136	0.017	0.703	**0.028**

**Having contracted at least one STD**	0.870	0.334	2.249	0.773

**Fear that positive HIV test would result in social exclusion**	1.214	0.596	2.483	0.592


## Discussion

In our study, which included 85 homeless people and 64 agricultural migrant workers living in Southern Italy, the cumulative prevalence of STI was high, and only half of the overall population returned to collect test results. Overall knowledge of STI modes of transmission was low and, in line with other studies [18,19], only 14% of the population reported consistent condom use.

In the present study, almost one homeless person out of ten reported having been engaged in chem-sex. This is concerning, because it is likely a proxy of the high rate of substance use disorder—and possibly also other mental health disorders—described in this and other marginalized populations [20]. Indeed, marginalization is correlated with increased high-risk behaviors [21] as well as higher disease burden and mortality [22]. In our population, the cumulative prevalence of STI was 24% and, interestingly, rates were similar among agricultural migrant workers (26.6%) and homeless people (23.5%), substantially exceeding the prevalence rates observed in the general population [23]. This data might be explained by the fact that the root causes of the disease burden lay in marginalization and social vulnerability themselves, rather than individual misconceptions or diverse cultural background [24], thus underscoring the intricate relationship between socio-economic vulnerability, substance abuse, barriers to healthcare, mental health, and the transmission of these viral infections.

Regarding the perceived stigma of HIV, the majority (59.1%) of the participants were afraid that a positive HIV test would result in social exclusion, and this conviction was likely supported by common misconception about disease route of transmission, as it is highlighted by the high rate of people who believed that HIV/AIDS might be contracted by sharing objects or by having meals together. This data, if combined with high STI prevalence might lead infected people to experience further social isolation, imprisoning them in a vicious circle of disease and marginalization.

Indeed, the main finding of our study was that, alarmingly, half of our population did not come back to collect test results, and the strongest predictor for not returning was having contracted HCV. This phenomenon highlights a critical gap in the healthcare system’s ability to engage and retain marginalized populations in the continuum of care. The situation is significantly exacerbated by the lack of consistent healthcare access, as evidenced by 69.1% of participants not having access to primary healthcare. The implications of this limited access are profound, contributing to delayed diagnosis and treatment, and consequently, to worse health outcomes and continued infection transmission [25].

For policymakers, this situation demands a reevaluation of current health policies and systems. It is crucial to implement strategies that not only increase healthcare accessibility but also build trust and engagement with marginalized communities. Furthermore, there is a demand for integrated care models that address not only the medical needs but also the social determinants of health, such as housing, employment, and mental health services [26]. These models can provide a more holistic approach to healthcare, addressing the root causes of healthcare disparities. Moreover, community-based interventions that involve peers or community leaders could significantly improve engagement and trust in healthcare services. This approach demonstrated to be effective in both migrant [27] and homeless [28] populations.

The present study has several limitations. Primarily, the generalizability of our findings is constrained by the study’s specific demographic and geographic focus on homeless individuals and agricultural migrant workers in Southern Italy. Secondly, our study’s reliance on self-reported data poses a risk of introducing bias, as participants’ responses may be influenced by recall and social desirability biases—including the potential underreporting of high-risk behaviors. Additionally, the cross-sectional design of the study inherently limits our ability to establish causality between the observed factors and the prevalence of STIs. Lastly, the absence of longitudinal follow-up in our study design precludes the assessment of long-term outcomes, which are crucial for understanding the progression of STIs and the effectiveness of interventions over time. These limitations highlight the need for further research with diverse populations and marginalized communities, preferably with longitudinal designs to explore the impact of interventions on health outcomes and retention in care.

## Conclusions

Among marginalized homeless and migrant communities, the prevalence of STIs and the failure to return to collect test results were high, and people who contracted HCV were more likely to be lost to follow-up. There is an urgent need to design interventions and policies that address vulnerable communities and explicitly target marginalization and socio-economic inequalities as key determinants of population health.

## Additional File

The additional file for this article can be found as follows:

10.5334/aogh.4388.s1 Supplementary file. Description of study sites and Supplementary Table 1.
