# Supplementary material for: Prevalence of Sexually Transmitted Infections and Predictors for Loss to Follow Up among Marginalized Homeless and Migrant Communities: a Cross-Sectional Study
Source: Ann Glob Health. 2024 Apr 1;90(1):25. doi: 10.5334/aogh.4388 (PMC11012089; doi:10.5334/aogh.4388)
Supplement: Supplementary file. — Description of study sites and Supplementary Table 1. [file agh-90-1-4388-s1.pdf]

Prevalence of sexually transmitted infections and predictors for loss to follow up among vulnerable homeless and migrant communities: a cross-sectional study.

Supplementary file

Description of study sites

- **Multifunctional Center for the fight against extreme poverty "Area 51":** low threshold daytime service, managed by the C.A.P.S. located in Corso Italia n. 79/81. The center provides a heterogeneous range of services (primary needs, legal and work counselling, and laboratory activities), in response to the main demands of homeless people or those in conditions of serious marginalization adult, promoting opportunities for socialization and professionalization.
- **"Andromeda" night center**: semi-residential night-time service, managed by the C.A.P.S., located in Corso Alcide De Gasperi n. 320/A, welcoming 44 homeless people (men and women), reported by the SSP of the Municipality of Bari, or placed, on an emergency basis, by the Social Emergency Service (managed by the C.A.P.S.), for a maximum period of 8 days. The center provides additional services aimed at responding to the primary needs of the guests welcomed (breakfast, access to toilets, personal hygiene kit) and promoting work reintegration and bureaucratic support interventions.
- **"Care for People" Street Unit:** itinerant mobile unit service, managed by the C.A.P.S., aimed at protecting people in conditions of serious adult marginalization present in the city area (through the monitoring of the areas in which homeless people usually live) and the implementation of interventions for the prevention of pathological addictions (through interventions to engage young people, in main places of aggregation and socialization). The service is active in the evening/night hours and makes use of a qualified team, coordinated by a social worker.
- **Community Houses**: low-threshold residential or semi-residential reception facilities, managed by various associations, operating in Bari or in other Municipalities of the Metropolitan City; the service can be carried out in housing units aimed at small families (minimum 6 people), or in accommodation with a maximum capacity of 120 places, with low care intensity. These places are for individuals or families in extreme poverty, or in conditions of serious socio-economic difficulty.
- **Pozzo Terraneo Settlement**: located in the countryside of Cerignola, it appears as an inhabited area, approximately 10 farmhouses, inhabited mostly by men coming mainly from Ghana, Gambia, and Senegal, engaged in agricultural activity. During the harvest periods there are around 600 people and then it gradually empties in the winter period, reaching around 100 people.

- **Ghetto Cicerone**: located in the countryside of San Marco in Lamis. It is a series of abandoned buildings occupied by around 300 labourers, mainly from Mali and Senegal. As for the other settlements, Cicerone estimates around 500 people in the harvest period to progressively empty in the winter months.
- **Casa Sankara:** It is an area in the San Severo countryside, organized and structured by the Apulia region that provides 400 beds with canteen service, air-conditioned housing modules with sanitation and it is located within the Fortore Agricultural Company.
- **Ghetto "L'Arena":** it is an informal settlement located in the San Severo countryside. These are two ruined buildings populated by around 200 young people from Senegal, Mali, Ghana, and Burkina Faso. There is no drinking water or electricity, the sanitary conditions are precarious, and it is a situation on the edge of legality.
- **Turi tent-city:** it is a shantytown that is created every year in the Turi countryside during the cherry harvest period. It consists of around 50 tents, without any sanitary facilities. It is populated by around 150-200 men, mainly from Egypt, Morocco, Tunisia, and sub-Saharan Africa.

Supplementary Table 1 – responses to HIV and HCV knowledge questionnaire

	Agricultural migrant workers (N=64)	Homeless people (N=85)	Overall (N=149)
**HIV can be transmitted by kissing/hugging a person living with HIV**			
Strongly agree	1 (1.6%)	6 (7.1%)	7 (4.7%)
Agree	23 (35.9%)	22 (25.9%)	45 (30.2%)
Neither agree nor disagree	30 (46.9%)	35 (41.2%)	65 (43.6%)
Disagree	7 (10.9%)	10 (11.8%)	17 (11.4%)
Strongly disagree	3 (4.7%)	12 (14.1%)	15 (10.1%)
**HIV is transmitted through unprotected sexual intercourse**			
Strongly agree	17 (26.6%)	29 (34.1%)	46 (30.9%)
Agree	19 (29.7%)	25 (29.4%)	44 (29.5%)
Neither agree nor disagree	24 (37.5%)	25 (29.4%)	49 (32.9%)
Disagree	4 (6.3%)	6 (7.1%)	10 (6.7%)
Strongly disagree	0 (0%)	0 (0%)	0 (0%)
**People can protect themselves from contracting HIV using condoms.**			
Strongly agree	11 (17.2%)	20 (23.5%)	31 (20.8%)
Agree	22 (34.4%)	28 (32.9%)	50 (33.6%)
Neither agree nor disagree	30 (46.9%)	33 (38.8%)	63 (42.3%)
Disagree	1 (1.6%)	3 (3.5%)	4 (2.7%)
Strongly disagree	0 (0%)	1 (1.2%)	1 (0.7%)
**HIV can be transmitted through blood transfusion or blood products.**			
Strongly agree	7 (10.9%)	20 (23.5%)	27 (18.1%)
Agree	13 (20.3%)	25 (29.4%)	38 (25.5%)
Neither agree nor disagree	38 (59.4%)	34 (40.0%)	72 (48.3%)
Disagree	6 (9.4%)	6 (7.1%)	12 (8.1%)
Strongly disagree	0 (0%)	0 (0%)	0 (0%)
**With HIV treatment, people can live a good quality of life and not be infectious.**			
Strongly agree	5 (7.8%)	13 (15.3%)	18 (12.1%)
Agree	5 (7.8%)	15 (17.6%)	20 (13.4%)
Neither agree nor disagree	45 (70.3%)	38 (44.7%)	83 (55.7%)
Disagree	9 (14.1%)	15 (17.6%)	24 (16.1%)
Strongly disagree	0 (0%)	4 (4.7%)	4 (2.7%)
**HIV is transmitted by sharing utensils and meals with people infected with HIV.**			
Strongly agree	2 (3.1%)	5 (5.9%)	7 (4.7%)
Agree	19 (29.7%)	26 (30.6%)	45 (30.2%)
Neither agree nor disagree	29 (45.3%)	27 (31.8%)	56 (37.6%)
Disagree	3 (4.7%)	13 (15.3%)	16 (10.7%)
Strongly disagree	5 (7.8%)	9 (10.6%)	14 (9.4%)
Strongly agree	6 (9.4%)	5 (5.9%)	11 (7.4%)
**HCV can be transmitted by kissing/hugging a person with HCV**			
Strongly agree	0 (0%)	3 (3.5%)	3 (2.0%)
Agree	4 (6.3%)	7 (8.2%)	11 (7.4%)
Neither agree nor disagree	53 (82.8%)	54 (63.5%)	107 (71.8%)
Disagree	4 (6.3%)	13 (15.3%)	17 (11.4%)
Strongly disagree	3 (4.7%)	8 (9.4%)	11 (7.4%)
**HCV causes liver cancer**			
Strongly agree	3 (4.7%)	7 (8.2%)	10 (6.7%)
Agree	4 (6.3%)	18 (21.2%)	22 (14.8%)
Neither agree nor disagree	52 (81.3%)	55 (64.7%)	107 (71.8%)
Disagree	3 (4.7%)	4 (4.7%)	7 (4.7%)
Strongly disagree	2 (3.1%)	1 (1.2%)	3 (2.0%)
**HCV is transmitted through sexual intercourse**			
Strongly agree	2 (3.1%)	8 (9.4%)	10 (6.7%)
Agree	8 (12.5%)	17 (20.0%)	25 (16.8%)
Neither agree nor disagree	45 (70.3%)	51 (60.0%)	96 (64.4%)

Disagree	3 (4.7%)	5 (5.9%)	8 (5.4%)
Strongly disagree	6 (9.4%)	4 (4.7%)	10 (6.7%)
**HCV is transmitted through contaminated blood**			
Strongly agree	6 (9.4%)	14 (16.5%)	20 (13.4%)
Agree	5 (7.8%)	23 (27.1%)	28 (18.8%)
Neither agree nor disagree	52 (81.3%)	44 (51.8%)	96 (64.4%)
Disagree	1 (1.6%)	3 (3.5%)	4 (2.7%)
Strongly disagree	0 (0%)	1 (1.2%)	1 (0.7%)
**There is a treatment that allows a cure for HCV**			
Strongly agree	3 (4.7%)	11 (12.9%)	14 (9.4%)
Agree	2 (3.1%)	9 (10.6%)	11 (7.4%)
Neither agree nor disagree	46 (71.9%)	46 (54.1%)	92 (61.7%)
Disagree	12 (18.8%)	14 (16.5%)	26 (17.4%)
Strongly disagree	1 (1.6%)	5 (5.9%)	6 (4.0%)
**HCV is transmitted by sharing utensils and meals with people infected with HCV**			
Strongly agree	5 (7.8%)	11 (12.9%)	16 (10.7%)
Agree	5 (7.8%)	17 (20.0%)	22 (14.8%)
Neither agree nor disagree	47 (73.4%)	38 (44.7%)	85 (57.0%)
Disagree	6 (9.4%)	13 (15.3%)	19 (12.8%)
Strongly disagree	1 (1.6%)	6 (7.1%)	7 (4.7%)
